# Supplementary material for: Investigating the Relationship Between Maternal Smoking During Pregnancy and Offspring Handedness: Extending the Proxy Gene-by-Environment Mendelian Randomization Study Design to Include Polygenic Risk Scores
Source: Behav Genet. 2025 Jul 16;55(4):289–301. doi: 10.1007/s10519-025-10226-0 (PMC12325536; doi:10.1007/s10519-025-10226-0)
Supplement: Supplementary file 1 — Supplementary file1 (DOCX 2281 KB) [file 10519_2025_10226_MOESM1_ESM.docx]

**Proxy Gene x Environment Regenie analysis**

One of the problems with GxE analyses in general is reduced statistical power. Stratifying the sample e.g. by maternal smoking status as we have done here, means that the number of individuals available for analysis is markedly reduced. Removing related individuals further reduces power, therefore we also ran proxy GxE genetic association analyses with related individuals included as sensitivity analyses. To do this we used Regenie [2] which is software developed to analyse binary traits with unbalanced case-control ratios on a genome-wide scale whilst also modelling population structure and cryptic relatedness. Results were largely unchanged when including related individuals in the analysis, whereby there was no association between the SNP rs16969968 and offspring hand preference (OR:1.02; 95% CI: 0.99, 1.06; p-value: 0.237).

**Power calculation**

We investigated the power of the proxy gene-by-environment Mendelian randomization study design to detect a causal effect of maternal smoking on risk of offspring left-handedness using data simulation. For all power calculations we tested the “reduced form” of the test statistic (i.e. the maternal SNP-offspring handedness association via logistic regression) in the subset of the cohort whose mothers smoked during pregnancy, rather than running a formal instrumental variables analysis. We simulated a single biallelic SNP (increaser allele frequency of q = 0.7) that explained 1% of the variance in number of cigarettes smoked per day by pregnant mothers, and subsequent transmission at this SNP from mothers and fathers to their offspring. For each offspring we then simulated a score on an underlying standard normal distribution of liability. Offspring who had scores above a threshold (corresponding to 10% of the population on the standard normal curve) were coded as being left-handed. We investigated two possible instrument strengths (maternal variants explained 1%, or 3% of the variance in cigarettes smoked per day), three possible sample sizes (total cohort size N = 500000, N = 1000000 and N = 2000000) and three different causal effect sizes (β_XY_ = 0.05, β_XY_ = 0.1, and β_XY_ = 0.2) which correspond to the degree to which one standard deviation increase in the number of cigarettes smoked per day (about 10 cigarettes in UK Biobank) increases the underlying liability of offspring being left-handed. For ease of interpretation, these causal effect sizes can be transformed into approximate odds ratios using the formula $OR\cong e^{\beta\times\frac{\pi}{\sqrt{3}}}$ and we include these approximations in our table of results. Offspring handedness was then regressed on the number of copies of their increaser allele at the locus. We assumed a type I error rate of α = 0.05 and performed 1000 replicates for each condition. Results are displayed in Supplementary Table 7 and our compute code is appended below. Our numbers assume that only ~30% of individuals would report having mothers that smoked during pregnancy.

#Script to calculate power to detect a causal effect using the proxy gene-by-environment Mendelian randomization study design

#Only simulate families where the mother smoked during pregnancy

#Assume that 30% of individuals have mothers who smoked during pregnancy

#Approximate odds ratio for 1 SD worth of smoking is exp(beta_xy*(pi/sqrt(3)))

rm(list=ls())

set.seed(12345)

Nrep = 1000 #Number of simulation replicates

N = 500000*0.3 #Number of individuals who smoked during pregnancy

p = 0.3 #Decreaser allele frequency

q <- 1-p #Increaser allele frequency

vg <- 0.03 #Variance in CPD explained by smoking SNP

beta_zx <- sqrt(vg)

beta_xy <- 0.2 #Causal effect of CPD on liability to left handedness. Measured in SDs.

prevalence <- 0.1 #Prevalence of left handedness

threshold <- qnorm(p=prevalence, mean = 0, sd = 1, lower.tail = FALSE, log.p = FALSE) #Threshold value for declaring an individual affected

alpha <- 0.05 #Significance level

vex <- 1 - beta_zx^2 #Residual variance in cigarettes per day

vey <- 1 - beta_xy^2 #Residual variance in liability to left handedness

pval <- vector(length = Nrep)

a <- sqrt(1/(2*p*q)) #Create genetic variable of variance one. Assume no dominance.

for(j in 1:Nrep) {

#Sample mothers' genotypes

Zm <- sample(x = c(-a,0,a), size = N, replace = TRUE, prob = c(p^2, 2*p*q, q^2))

#Sample fathers' genotypes

Zf <- sample(x = c(-a,0,a), size = N, replace = TRUE, prob = c(p^2, 2*p*q, q^2))

Zo <- vector(length = N)

#Simulate offspring genotype

r <- runif(N)

for (i in 1:N) { #This is not efficient...

if((Zm[i]==-a) && (Zf[i]==-a)) {Zo[i] = -a}

if((Zm[i]==-a) && (Zf[i]==0)) {if(r[i] <= 0.5) {Zo[i] = -a} else {Zo[i] = 0}}

if((Zm[i]==-a) && (Zf[i]==a)) {Zo[i] = 0}

if((Zm[i]==0) && (Zf[i]==-a)) {if(r[i] <= 0.5) {Zo[i] = -a} else {Zo[i] = 0}}

if((Zm[i]==0) && (Zf[i]==0)) {

if(r[i] <= 0.25) {Zo[i] = -a}

if(r[i] > 0.25 && r[i] <= 0.75) {Zo[i] = 0}

if(r[i] > 0.75) {Zo[i] = a}

}

if((Zm[i]==0) && (Zf[i]==a)) {if(r[i] <= 0.5) {Zo[i] = a} else {Zo[i] = 0}}

if((Zm[i]==a) && (Zf[i]==-a)) {Zo[i] = 0}

if((Zm[i]==a) && (Zf[i]==0)) {if(r[i] <= 0.5) {Zo[i] = a} else {Zo[i] = 0}}

if((Zm[i]==a) && (Zf[i]==a)) {Zo[i] = a}

}

#Simulate maternal number of cigarettes

X_m <- beta_zx*Zm + rnorm(N,0,sqrt(vex))

#Simulate underlying distribution of liability to left handedness

Y_l <- beta_xy*X_m + rnorm(N,0,sqrt(vey))

Y_o <- as.integer(Y_l > threshold)

result <- glm(Y_o ~ Zo, family='binomial')

pval[j] <- summary(result)$coefficients[2,4]

}

power <- sum(as.integer(pval < alpha))/Nrep

power

li_power <- power - 1.96*sqrt((power/(1-power))/Nrep)

li_power

hi_power <- power + 1.96*sqrt((power/(1-power))/Nrep)

hi_power

#Compare logistic beta approximation to simulated logistic coefficient

beta_xy*(pi/sqrt(3))

summary(glm(Y_o ~ X_m, family='binomial'))

#Approximate odds ratio per 1SD CPD

OR<-exp(beta_xy*(pi/sqrt(3)))

OR

| **Supplementary Table 1: Variables included in analysis and coding information** | | | |
| --- | --- | --- | --- |
|  | UK Biobank code | Type | Additional information |
| Year of Birth | f.34.0.0 | Continuous | 1936-1971 |
| Birthweight | f.20022.0.0 | Continuous | 0.45-5.98 (kg) |
| Part of a multiple birth | f.1777.0.0 | Categorical | 0=No, 1=Yes |
| Breastfed | f.1677.0.0 | Categorical | 0=Yes, 1=No |
| Maternal smoking during pregnancy (reported by offspring) | f.1787.0.0 | Categorical | 0=No, 1=Yes |
| Country of Birth | f.1647.0.0 | Categorical | England treated as baseline (0=England), with all other countries coded by a series of dichotomous variables. |
| Sex | f.31.0.0 | Categorical | 0=Female, 1=Male |
| Month of birth | f.52.0.0 | Continuous | Modelling using a cosine function for the 12 months |
| Handedness |  | Categorical | 0=right-handed, 1= left-handed, 2=uses both hands equally |

| **Supplementary Table 2: Variants used to construct the PRS, their closest gene and putative function** | | | | | | | | | | | |  | |
| --- | --- | --- | --- | --- | --- | --- | --- | --- | --- | --- | --- | --- | --- |
| **CHR** | **POS** | **RSID** | **EA** | **OA** | **EAF** | **Beta** | **SE** | **P** | **N** | **Closest Gene** | **Putative gene Function** | **Known association** |  |
| 8 | 27568560 | rs1565735 | A | T | 0.20 | 0.02 | 0.004 | 4.33x10^-8^ | 183196 | intergenic |  | Smoking cessation, smoking status, smoking initiation, cigarettes per day, height |  |
| 9 | 15812667 | rs10962170 | G | A | 0.43 | 0.02 | 0.003 | 1.71x10^-8^ | 183196 | *CCDC171* | DNA-binding transcription factor activity and obsolete signal transducer activity |  |  |
| 11 | 113448730 | rs4274224 | A | G | 0.55 | 0.02 | 0.003 | 2.68x10^-8^ | 183196 | *DRD2* | Synthesis regulation, storage, and release of dopamine |  |  |
| 11 | 46741267 | rs61884324 | A | G | 0.10 | 0.03 | 0.006 | 2.59x10^-9^ | 183196 | *F2, CKAP5* | Regulates microtubule chromosome attachments, encodes prothrombin protein | smoking initiation |  |
| 15 | 78559273 | rs2036527 | A | G | 0.37 | 0.10 | 0.003 | 2.1x10^-17^ | 183196 | *CHRNA5* | Affects response dopaminergic neurons during chronic nicotine exposure and withdrawal | Average tendency to become a heavy smoker, response to bronchodilator (medication that relieves the symptoms of asthma), pulmonary function measurement, forced expiratory volume, cotinine measurement |  |
| 16 | 52063640 | rs11076320 | C | A | 0.63 | 0.02 | 0.003 | 8.88x10^-10^ | 181256 | *LINC02911* | Intergenic non-protein coding |  |  |
| 16 | 89827742 | rs1108064 | G | C | 0.57 | 0.02 | 0.003 | 6.46x10^-10^ | 181256 | *SPIRE2* | Establishment of meiotic spindle localization |  |  |
| 17 | 29236995 | rs112178027 | T | C | 0.18 | 0.03 | 0.004 | 1.52x10^-8^ | 183196 | *CRYBA1, TWF1P1* | Protein homodimerization activity and structural constituent of eye lens | Platelet count, brain stem volume measurement |  |
| 19 | 40743318 | rs142449067 | C | T | 0.04 | 0.05 | 0.01 | 2.44x10^-8^ | 183073 | *C19ORFf54* |  |  |  |
| 20 | 32361562 | rs6141293 | C | T | 0.35 | 0.02 | 0.003 | 4.95x10^-12^ | 183196 | *ASXL1* | Involved in chromatin remodelling |  |  |
| 20 | 63355597 | rs2273500 | C | T | 0.15 | 0.04 | 0.005 | 9.06x10^-16^ | 176936 | *CHRNA4* | Involved in the synthesis of neuronal nictotinic acetylecholine receptor. | Cigarettes per day, nicotine dependence, parental longevity, chronic obstructive pulmonary disease |  |
| 21 | 39272184 | rs145104523 | T | C | 0.13 | 0.03 | 0.005 | 4.18x10^-8^ | 183196 | *BRWD1* | Epigenetic control of meiotic chromosome stability in females and haploid gene transcription during post meiotic sperm differentiation in males. | Intelligence, BMI, ADHD, ASD |  |
| *Nb: SNPs presented here were used to construct the PRS. Beta refers to the increase in the number of cigarettes smoked per day per increaser allele, SE refers to standard error, EA refers to the effect allele, OA refers to the other allele (reference allele), p refers to the p-value, N describes the sample size across contributing cohorts.* | | | | | | | | | | | | | |
|  |  |  |  |  |  |  |  |  |  |  |  |  |  |
|  |  |  |  |  |  |  |  |  |  |  |  |  |  |

| **Supplementary Table 3: Distribution of observed characteristics at baseline assessment in UK Biobank for participants who have no missing phenotype values.** | | | |
| --- | --- | --- | --- |
| **Variable** | **Categories** | **N (mean for continuous variables and % for categorical variables)** | **Frequency of left-hand preference vs righthanded (%)** |
| **Sex** | Female | 119, 903 (62.08) | 8.90 |
|  | Male | 73252 (37.92) | 10.98 |
| **Part of a multiple birth** | No | 188,171 (97.42) | 9.64 |
|  | Yes | 4984 (2.58) | 11.50 |
| **Maternal smoking** | No | 136, 946 (70.90) | 9.80 |
|  | Yes | 56, 209 (29.10) | 9.42 |
| **Breastfed** | No | 58, 692 (30.39) | 10.35 |
|  | Yes | 134,463 (69.61) | 9.40 |
| **Birthweight** |  | 193,155 (3.33 kg) |  |
| **Birth Year** |  | 193,155 (1953) |  |
| **Birth Month** |  | 193,155 (June) |  |
| **Social deprivation index** |  | 193,155 (-1.64) |  |
| **Country of Origin** | England | 162,578 (84.17) | 10.04 |
|  | Wales | 9563 (4.95) | 7.03 |
|  | Scotland | 16333 (8.46) | 7.92 |
|  | Northern Ireland | 862 (0.44) | 8.35 |
|  | Republic of Ireland | 77 (0.04) | 7.79 |
|  | Elsewhere | 3742 (1.94) | 9.35 |
| *NB: Maternal smoking during pregnancy was reported by offspring.* | | | |

| **Supplementary Table 4**: Associations between predictor variables and handedness (right-handedness=0, left-handedness=1) | | | | | | | | | | | | | | |
| --- | --- | --- | --- | --- | --- | --- | --- | --- | --- | --- | --- | --- | --- | --- |
|  | Year of Birth | Birthweight | Part of a multiple birth  (0=No, 1=Yes) | Breastfed  (0=Yes, 1=No) | Maternal Smoking  (0=No, 1=Yes) | Month of birth (cosine) | Sex  (0=Female, 1=Male) | England | Wales | Scotland | Northern Ireland | Republic of Ireland | Elsewhere | Handedness |
| Year of Birth | 1 |  |  |  |  |  |  |  |  |  |  |  |  |  |
| Birthweight | 0.006* | 1 |  |  |  |  |  |  |  |  |  |  |  |  |
| Part of a multiple birth | -0.013** | -0.178** | 1 |  |  |  |  |  |  |  |  |  |  |  |
| Breastfed | 0.175** | -0.081** | 0.053 | 1 |  |  |  |  |  |  |  |  |  |  |
| Maternal smoking | 0.012** | -0.069** | 0.00002 | 0.090 | 1 |  |  |  |  |  |  |  |  |  |
| Month of birth (cosine) | 0.009 | -0.020** | -0.001 | 0.011** | 0.003 | 1 |  |  |  |  |  |  |  |  |
| Sex | 0.006* | 0.153** | 0.006* | 0.039** | 0.012** | 0.0002 | 1 |  |  |  |  |  |  |  |
| England | -0.006* | 0.009 ** | 0.003** | 0.030 | 0.020 | 0.001 | 0.022 | 1 |  |  |  |  |  |  |
| Wales | -0.025** | -0.002 | 0.002** | 0.032 | 0.003 | -0.004 | 0.002 | NA | 1 |  |  |  |  |  |
| Scotland | -0.028** | 0.006* | 0.002** | 0.061** | 0.027** | 0.001 | 0.009 | NA | NA | 1 |  |  |  |  |
| Northern Ireland | 0.0009 | 0.004 | 0.0003 | 0.022** | 0.001 | 0.001 | 0.0009 | NA | NA | NA | 1 |  |  |  |
| Republic of Ireland | -0.012** | 0.004 | 0.009** | 0.024** | 0.005* | -0.003 | 0.005 | NA | NA | NA | NA | 1 |  |  |
| Elsewhere | 0.065** | -0.023** | 0.009** | 0.060** | 0.066** | 0.005* | 0.025** | NA | NA | NA | NA | NA | 1 |  |
| Handedness | 0.017** | -0.003 | 0.010** | 0.017** | 0.004 | -0.006 | 0.034** | 0.036** | 0.019** | 0.019** | 0.002 | 0.004 | 0.020** | 1 |
| For associations between categorical variables, Cramer’s V is presented. Associations between continuous variables are shown as Pearson R. Associations between categorical and continuous variables are shown as Spearman rho.  * = P < 0.05  ** = P < 0.001 | | | | | | | | | | | | | | |

| **Supplementary Table 5: Distribution of observed characteristics at baseline assessment in UK Biobank participants** | | | | | |
| --- | --- | --- | --- | --- | --- |
|  |  | **Mother smoked during pregnancy** | | **Mother who did not smoke during pregnancy** | |
| **Variable** | **Categories** | **N (mean for continuous variables and % for categorical variables)** | **Frequency of left-hand preference (%)** | **N (mean for continuous variables and % for categorical variables)** | **Frequency of left-hand preference (%)** |
| Number of smoking heaviness increasing alleles | 0 | 62903 | 9.81 | 115,332 | 9.94 |
|  | 1 | 62206 | 9.81 | 112,837 | 9.94 |
|  | 2 | 15410 | 9.67 | 27,616 | 9.47 |
| Sex | Male | 73,763 | 11.03 | 116,784 | 9.94 |
|  | Female | 66,756 | 8.68 | 139,001 | 8.95 |
| Year of birth |  | 140,519 (1951.37) |  | 255,785  (1950.99) |  |
| Social deprivation |  | 140,359  (-1.29) |  | 140,359  (-1.65) |  |
| *NB: Maternal smoking during pregnancy was reported by offspring.* | | | | | |

| **Supplementary Table 6.1: Association between rs16969968 and weighted PRS for smoking heaviness and binary predictor variables** | | | | | | | | | |
| --- | --- | --- | --- | --- | --- | --- | --- | --- | --- |
|  |  | **Whole cohort** | | | | **Maternal smoking during pregnancy** | | | |
| **Threshold** | **exposure** | **OR** | **95% CI** | ***p*** | **R^2^** | **OR** | **95% CI** | ***p*** | **R^2^** |
| rs16969968 | Maternal smoking during pregnancy | 1.02 | 1.01, 1.03 | 3.5x10^-4^ | 3.5x10^-5^ |  |  |  |  |
|  | Sex | 0.996 | 0.986, 1.01 | 0.412 | 1.4x10^-6^ | 0.990 | 0.974, 1.01 | 0.217 | 8.33x10^-6^ |
|  | Breastfed | 1.00 | 0.988, 1.01 | 0.902 | 1.5x10^-8^ | 0.999 | 0.979, 1.02 | 0.887 | 7.8x10^-7^ |
|  | Part of a multiple birth | 1.03 | 0.992, 1.06 | 0.132 | 3.5x10^-5^ | 1.068 | 1.01, 1.12 | 0.017 | 2.0x10^-3^ |
|  | Maternal smoking during pregnancy | 1.865 | 1.65, 2.08 | 1.37x10^-8^ | 7.13x10^-3^ |  |  |  |  |
|  | Sex | 0.786 | 0.602, 0.969 | 0.010 | 1.20x10^-4^ | 0.722 | 0.435, 1.01 | 0.026 | 1.66x10^-4^ |
|  | Breastfed | 0.962 | 0.729, 1.19 | 0.745 | 5.74x10^-3^ | 1.02 | 0.660, 1.39 | 0.898 | 5.12x10^-3^ |
|  | Part of a multiple birth | 2.511 | 1.88, 3.15 | 0.004 | 4.39x10^-4^ | 4.18 | 3.19, 5.16 | 0.005 | 7.2x10^-4^ |
| **Supplementary Table 6.2: Association between rs16969968 and PRS for smoking heaviness and continuous predictor variables** | | | | | | | | | |
|  | **exposure** | **beta** | **95% CI** | ***p*** | **R^2^** | **beta** | **95% CI** | ***p*** | **R^2^** |
| rs16969968 | Offspring smoking heaviness | 0.863 | 0.771, 0.955 | <2x10^-16^ | 0.00516 | 0.884 | 0.744, 1.024 | <2x10^-16^ | 0.00540 |
|  | Birthweight | -0.006 | -0.01, -0.001 | 0.01 | -3.2x10^-6^ | -0.012 | -0.02, -0.005 | 0.001 | 0.0001 |
|  | Year of Birth | 0.009 | -0.031, 0.049 | 0.662 | -2.0x10^-6^ | 0.064 | 0.003, 0.125 | 0.039 | 2.49x10^-5^ |
|  | Social deprivation | 0.002 | -0.013, 0.017 | 0.778 | -1.4x10^-6^ | -0.012 | -0.036, 0.012 | 0.341 | -3.56x10-6 |
|  | Birth month (cosine) | 0.001 | -0.002, 0.005 | 0.552 | -1.87x10^-6^ | -0.002 | -0.008, 0.003 | 0.437 | -3.24x10^-6^ |
| 5x10^-8^ | Offspring smoking heaviness | 16.914 | 15.234, 18.594 | <2x10^-16^ | 0.00579 | 16.621 | 14.065, 19.178 | <2x10^-16^ | 0.00565 |
|  | Birthweight | -0.112 | -0.189, -0.034 | 0.005 | 0.001 | -0.20 | -0.334, -0.071 | 0.003 | 0.001 |
|  | Year of Birth | 0.477 | -0.256, 1.21 | 0.202 | 0.003 | 1.60 | 0.492, 2.71 | 0.005 | 0.004 |
|  | Social deprivation | 0.277 | 0.01, 0.546 | 0.042 | 0.013 | 0.201 | -0.237, 0.639 | 0.368 | 0.012 |
|  | Birth month (cosine) | 0.052 | -0.012, 0.117 | 0.112 | 3.74x10^-5^ | -0.004 | -0.106, 0.097 | 0.933 | -1.76x10^-5^ |
| *NB: All associations adjusted for the first ten genetic principal components. The difference in size of the OR/beta between the single SNP and the PRS is partly due to the PRS being weighted. Maternal smoking during pregnancy was reported by offspring. Offspring smoking heaviness is reported by number of cigarettes smoked per day with outliers (3sd above or below the mean) removed.* | | | | | | | | | |

| **Supplementary Table 7: Statistical power to detect a causal effect of maternal smoking on offspring left-handedness using the reduced form equation across varying causal effect sizes, sample sizes and instrument strengths (N=1000 replicates per condition).** | | | | | | | | | | | | | | | | | | |
| --- | --- | --- | --- | --- | --- | --- | --- | --- | --- | --- | --- | --- | --- | --- | --- | --- | --- | --- |
|  | **Sample size: 500000** | | | | | | **Sample size: 1000000** | | | | | | **Sample size: 2000000** | | | | | |
| **Causal effect** | **β_XY_ = 0.05**  **(OR = 1.09)** | | **β_XY_ = 0.1**  **(OR = 1.2)** | | **β_XY_ = 0.2**  **(OR = 1.44)** | | **β_XY_ = 0.05**  **(OR = 1.09)** | | **β_XY_ = 0.1**  **(OR = 1.2)** | | **β_XY_ = 0.2**  **(OR = 1.44)** | | **β_XY_ = 0.05**  **(OR = 1.09)** | | **β_XY_ = 0.1**  **(OR = 1.2)** | | **β_XY_ = 0.2**  **(OR = 1.44)** | |
|  | **Pwr** | **95% CI** | **Pwr** | **95% CI** | **Pwr** | **95% CI** | **Pwr** | **95% CI** | **Pwr** | **95% CI** | **Pwr** | **95% CI** | **Pwr** | **95% CI** | **Pwr** | **95% CI** | **Pwr** | **95% CI** |
| Instrument strength 1% | 0.069 | 0.053, 0.085 | 0.173 | 0.150, 0.196 | 0.644 | 0.614, 0.674 | 0.133 | 0.112, 0.154 | 0.361 | 0.331, 0.391 | 0.884 | 0.864, 0.904 | 0.177 | 0.153, 0.201 | 0.588 | 0.557, 0.619 | 0.990 | 0.984, 0.996 |
| Instrument strength 3% | 0.169 | 0.146, 0.192 | 0.514 | 0.483, 0.545 | 0.968 | 0.957, 0.979 | 0.283 | 0.255, 0.311 | 0.814 | 0.790, 0.838 | 1.00 | 1.000, 1.000 | 0.477 | 0.446, 0.508 | 0.988 | 0.981, 0.995 | 1.00 | 0.996, 1.004 |
| Pwr = Power.  95% CI = Monte Carlo 95% confidence interval.  β_XY_ = Causal effect of maternal smoking on offspring liability to left handedness. Betas refer to the expected increase in standard normal liability to left handedness per standard deviation increase in number of cigarettes smoked per day during pregnancy.  OR = Approximate odds ratio for left handedness corresponding to a standard deviation increase in number of cigarettes smoked per day during pregnancy. | | | | | | | | | | | | | | | | | | |

UKB Cohort (N=502,512)

Excluded individuals:

Withdrawn consent: 52

Individuals not of White European ancestry: 59,910

Individuals of White European ancestry only

(N=442,550)

Individuals with birthweight between 1kg and 6kg (441,301)

Excluded individuals:

Individuals that report ambidexterity: 7105

Individuals who did not report hand preference: 92

Excluded individuals:

Individuals with birthweight greater than 6kg: 236

Individuals with birthweight less than 1kg: 1013

Left-handed and right -handed individuals only (N=434,104)

**Figure 1: Flow diagram of exclusion criteria for traditional observational analyses**

Excluded individuals:

Aneuploidy: 631

Excluded from Kinship: 787

Heterozygosity outliers: 968

Sex mismatch: 166

(Some individuals fall into more than one category. Total individuals removed: 1493)

**Figure 2: Flow diagram of exclusion criteria for proxy GxE MR analyses**

Unrelated left-handed and right -handed individuals with genetic QC (N=346,871)

UKB Cohort (N=502,512)

Excluded individuals:

Withdrawn consent: 52

Individuals not of White European ancestry: 59,910

Individuals of White European ancestry only

(N=442,550)

Individuals with birthweight between 1kg and 6kg (441,301)

Excluded individuals:

Individuals that report ambidexterity: 7105

Individuals who did not report hand preference: 92

Left-handed and right -handed individuals only (N=434,104)

Excluded individuals:

Individuals with birthweight greater than 6kg: 236

Individuals with birthweight less than 1kg: 1013

Left-handed and right -handed individuals with genetic QC (N=432,519)

Excluded individuals:

Related individuals: 81,369

No genetic data [1]: 4279

**Figure 3: Association between early-life predictors of handedness and left-hand preference in females only.**

An odds ratio > 1 indicates propensity to be left-handed. Unadjusted refers to results from univariable logistic regression analyses. Adjusted refers to results from multivariable regression analyses.

*NB: Maternal smoking during pregnancy was reported by offspring.*


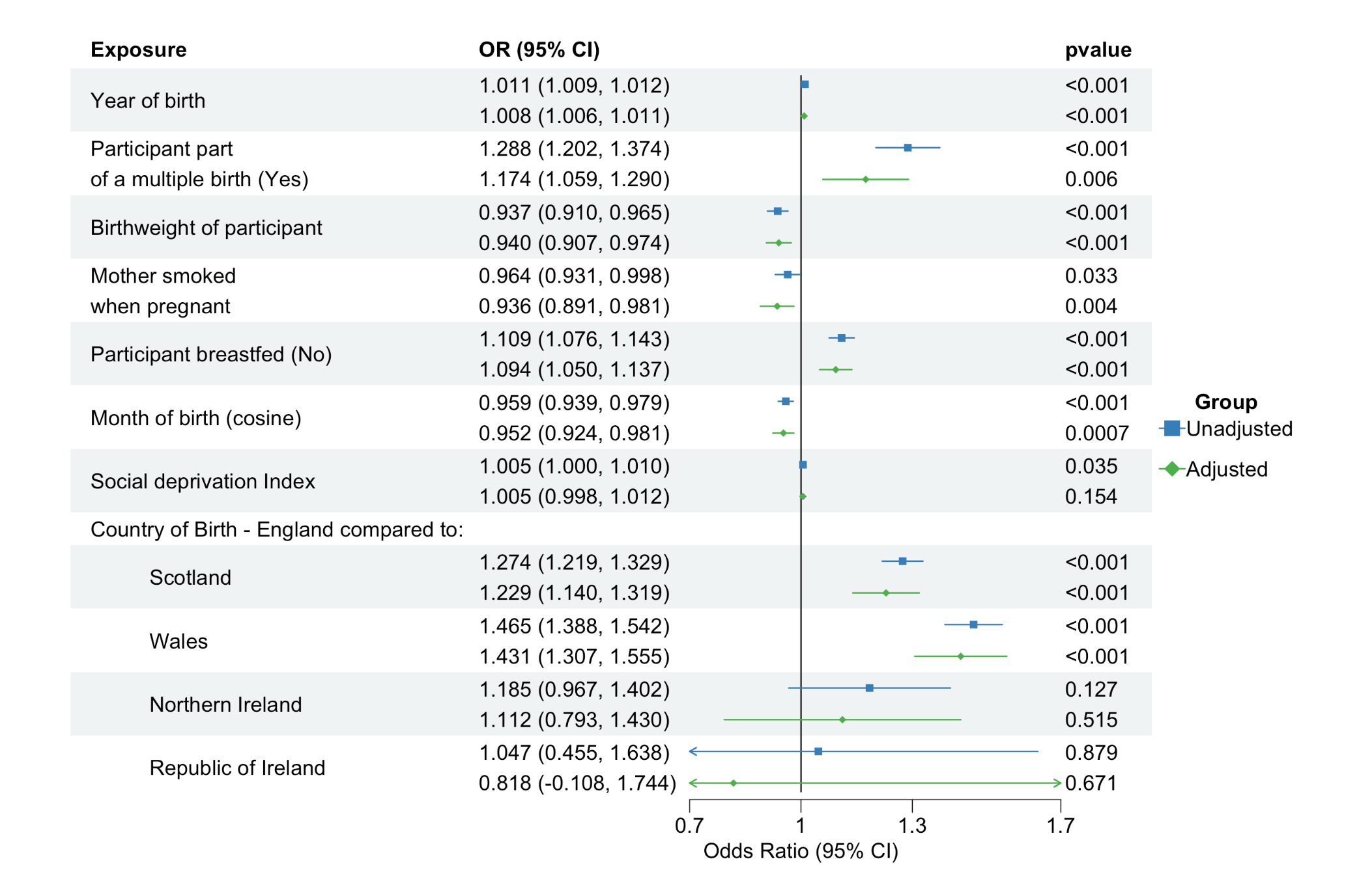


**Figure 4: Association between early-life predictors of handedness and left-hand preference in males only.**

An odds ratio > 1 indicates propensity to be left-handed. Unadjusted refers to results from univariable logistic regression analyses. Adjusted refers to results from multivariable regression analyses.

*NB: Maternal smoking during pregnancy was reported by offspring.*


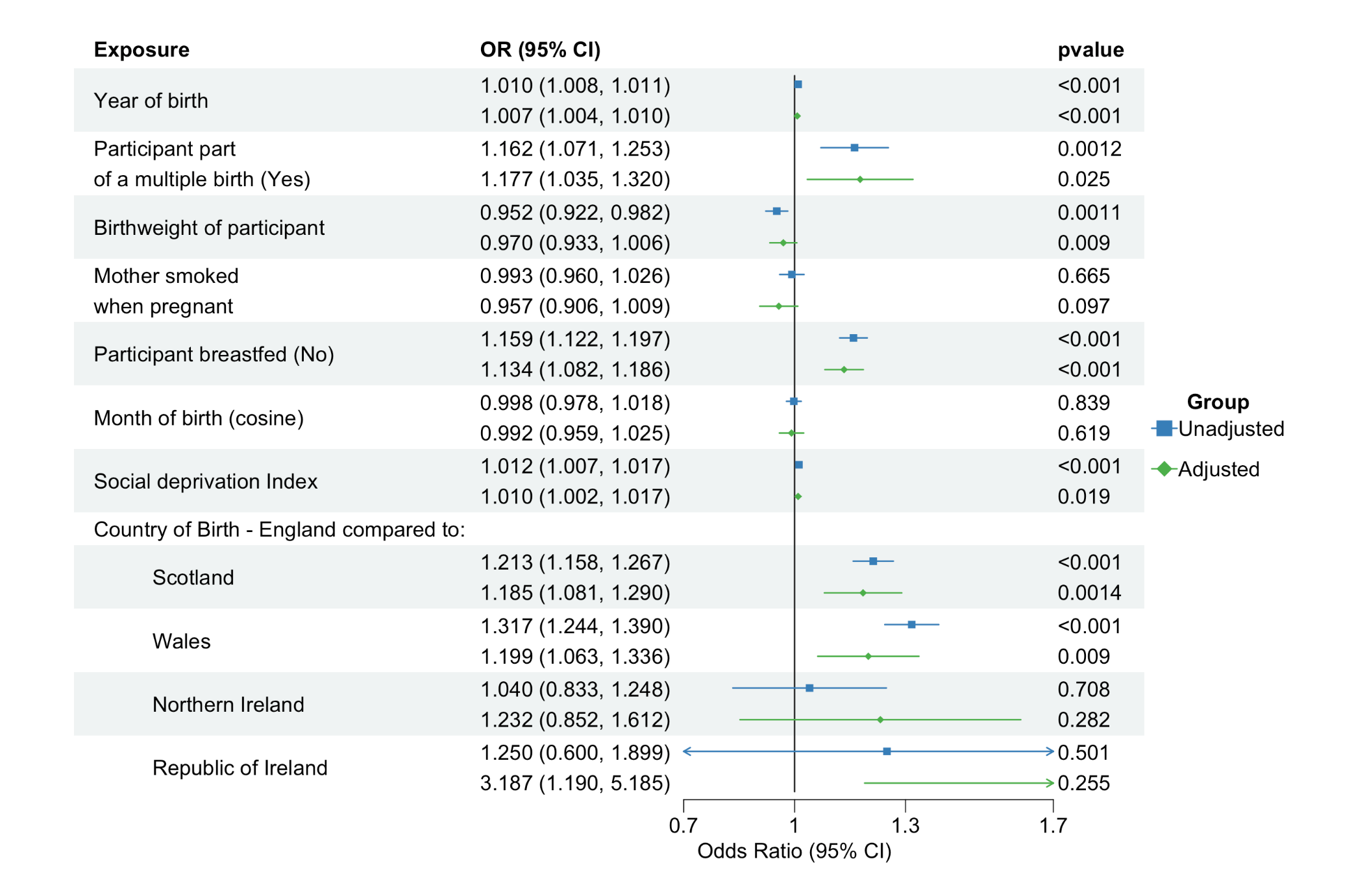


1. Bycroft, C., et al., *The UK Biobank resource with deep phenotyping and genomic data.* Nature, 2018. **562**(7726): p. 203-209.

2. Mbatchou, J., et al., *Computationally efficient whole-genome regression for quantitative and binary traits.* Nature Genetics, 2021. **53**(7): p. 1097-1103.
